# Cinnamaldehyde and baicalin reverse colistin resistance in *Enterobacterales* and *Acinetobacter baumannii* strains

**DOI:** 10.1007/s10096-024-04884-x

**Published:** 2024-07-27

**Authors:** Natalia A. Mireles, Cristina F. Malla, María M. Tavío

**Affiliations:** 1 Microbiology, Clinical Science Department, Faculty of Health Sciences, Universidad de Las Palmas de Gran Canaria, Paseo Blas Cabrera Felipe s/n, Las Palmas de Gran Canaria, 35016 Spain; 2 Present Address: Medical Oncology, Josep Trueta University Hospital of Girona, Girona, 17007, Spain

**Keywords:** Cinnamaldehyde, Baicalin, Colistin-resistance, Gram-negative bacteria, *Cinnamomum*, *Scutellaria*

## Abstract

**Purpose:**

Colistin is used as a last resort antibiotic against infections caused by multidrug-resistant gram-negative bacteria, especially carbapenem-resistant bacteria. However, colistin-resistance in clinical isolates is becoming more prevalent. Cinnamaldehyde and baicalin, which are the major active constituents of *Cinnamomum* and *Scutellaria*, have been reported to exhibit antibacterial properties. The aim of this study was to evaluate the capacity of cinnamaldehyde and baicalin to enhance the antibiotic activity of colistin in *Enterobacterales* and *Acinetobacter baumannii* strains.

**Methods:**

The MICs of colistin were determined with and without fixed concentrations of cinnamaldehyde and baicalin by the broth microdilution method. The FIC indices were also calculated. In addition, time-kill assays were performed with colistin alone and in combination with cinnamaldehyde and baicalin to determine the bactericidal action of the combinations. Similarly, the effects of L-arginine, L-glutamic acid and sucrose on the MICs of colistin combined with cinnamaldehyde and baicalin were studied to evaluate the possible effects of these compounds on the charge of the bacterial cell- wall.

**Results:**

At nontoxic concentrations, cinnamaldehyde and baicalin partially or fully reversed resistance to colistin in *Enterobacterales* and *A. baumannii*. The combinations of the two compounds with colistin had bactericidal or synergistic effects on the most resistant strains. The ability of these agents to reverse colistin resistance could be associated with bacterial cell wall damage and increased permeability.

**Conclusion:**

Cinnamaldehyde and baicalin are good adjuvants for the antibiotic colistin against *Enterobacterales*- and *A. baumannii*-resistant strains.

## Introduction

Antimicrobial resistance is considered a major threat to global health. There has been particular attention on multidrug-resistant (MDR) pathogens from the genera *Acinetobacter, Pseudomonas*, *Klebsiella*, *Enterobacter*, *Serratia* and *Proteus*, and the species *Escherichia coli*, which pose an important clinical threat and are responsible for severe and often deadly infections [1–3]. In this context, interest in older antibiotics, such as colistin, has resurfaced. Colistin is a polymyxin antibiotic that is used as a last-resort treatment for life-threatening infections caused by gram-negative MDR bacteria [4, 5]. Colistin functions mainly by interacting with lipopolysaccharide (LPS), which is part of the outer membrane of gram-negative bacteria. Once attached, colistin causes derangement and lysis of the cell wall and the leakage of internal contents [5].

Unfortunately, the extensive use of colistin has led to the spread of both chromosomally and plasmid-mediated colistin resistance. However, acquired chromosomal resistance to colistin remains the most common in *Enterobacterales* strains isolated from humans [5, 6]. In addition, species such as *Serratia marcescens*, *Proteus* spp., *Providencia* spp. and *Morganella morganii* exhibit intrinsic resistance to this antibiotic [5]. To address the increasing emergence and spread of antibiotic resistance, promoting new solutions that broaden the antimicrobial portfolio is important. Quorum sensing (QS) inhibitors have emerged as possible alternatives to antibiotics [7].

Cinnamaldehyde (CN) is a flavonoid that gives cinnamon its flavor and aroma [8]. The synergistic activity of CN with antibiotics, effects on biofilms and QS regulation have been demonstrated [9, 10]. CN is broadly used in the food and cosmetic industries [8, 9].

Baicalin (BA) is also a flavonoid that is mainly found in the roots of plants of the genus *Scutellaria*. BA enhances antibiotic activity and acts as QS inhibitor [11, 12]. BA also inhibits the production of biofilms, and other virulence factors in *Pseudomonas aeruginosa* and *E. coli* [11, 12].

The aim of this study was to assess the potential of using CN and BA to enhance colistin activity against *Enterobacterales* and *Acinetobacter baumannii*.

## Materials and methods

### Strains

This study involved fifteen colistin-resistant clinical bacterial isolates of the species *Klebsiella pneumoniae* (*n* = 6), *E. coli* (*n* = 3), *Enterobacter cloacae* (*n* = 3), *S. marcescens* (*n* = 1), *Proteus mirabilis* (*n* = 1), and *M. morganii* (*n* = 1), which were all isolated from blood samples in 2019 (strains 19/) or 2021 (strains 21/) and differentiated by biotype using the API20E biochemical test and database at https://apiweb.biomerieux.com/. These strains were also differentiated by antibiotype via the determination of beta-lactam MICs using Sensititre™ EU Surveillance ESBL EUVSEC2 AST plates (Thermo Fisher Scientific) and colistin and ciprofloxacin MICs. This study also included five wild-type reference strains from the Spanish Collection of Type Cultures (*E. coli* ATCC 25922, *K. pneumoniae* ATCC 700603, *P. aeruginosa* ATCC 27853, *A. baumannii* ATCC 15308 and *A. baumannii* ATCC 19606) and two stable colistin-resistant mutants of ATCC 15308 and ATCC 19606, which were selected in vitro as previously described [13]. The selection frequencies of the two mutants were consistent with those reported in a previous study [13].

All clinical isolates were extended-spectrum beta-lactamase (ESBL)-producing strains according to EUCAST guidelines, which are based on non-susceptibility to indicator oxyimino-cephalosporins; all the strains were also MDR strains according to Magiorakos et al. since they were beta-lactam-, quinolone- and colistin-resistant [14, 15]. The colistin- resistance in strains 19/2, 19/3 to 19/8 and 19/10 to 19/12 was previously characterized as chromosomally- mediated [16].

### Identification of plasmid-mediated resistance to colistin

The presence of the *mcr-1* to *mcr-10* genes was studied in fifteen colistin-resistant clinical bacterial isolates, including the 19/2, 19/3 to 19/8 and 19/10 to 19/12 strains, as previously described [17–20]. PCR of the studied strains was performed on genomic DNA, which was extracted by the boiling method, and on plasmid DNA, which was extracted by using a High Pure Plasmid Isolation Kit from Roche.

In addition, the transferability of colistin resistance was assessed by conjugation assays using the sodium-azide-resistant *E. coli* J53 K12 strain as a recipient [16].

### Minimum inhibitory concentration (MIC) assessment and fractional inhibitory concentration (FIC) index calculation

The MICs of colistin, CN and BA, which were purchased from Sigma (Spain), were determined individually as previously described [16]. Colistin was dissolved in water [16]. Furthermore, ethanol and dimethyl sulfoxide (DMSO) were used as solvents for CN and BA, respectively.

The range of concentrations of colistin was from 0.063 to 4096 mg/L. The MICs of colistin were determined in the presence of fixed concentrations of CN (28.5, 57 and 114 mg/L) at pH 7.0- 7.4 and BA (1.3 × 10^3^, 2.7 × 10^3^ and 5.4 × 10^3^ mg/L) at pH 5.2–6.34. These concentrations of CN and BA were 1/2, 1/4 and 1/8 of the MICs of these chemicals in the studied strains, except for *A. baumannii* 21/7, in which the BA MIC was 1/2 that in the remaining strains studied. The MICs of CN and BA were in the range of previous descriptions [12, 21].

MICs were determined by the 2-fold broth microdilution method in Mueller-Hinton broth (MHB) according to the latest EUCAST and CLSI guidelines [22–24]. All MIC determinations were performed at least three times.

Fractional inhibitory concentration indices (FICIs) were calculated to assess the synergy between colistin and the two agents. The same units were used for all MIC values to calculate the FICIs as follows: FICI = FICA + FICB, where FICA = MICA + B/MICA, and FICB = MICA + B/MICB, in this equation, A was colistin and B was cinnamaldehyde or baicalin. An FICI ≤ 0.5 suggested synergy, an FICI > 0.5-4 suggested no interaction, and an FICI > 4.0 suggested antagonism [25].

### Time-kill assay

Time-kill assays were performed on six strains with the highest colistin resistance (colistin MICs ≥ 128 mg/L), *E. cloacae* 19/3, *K. pneumoniae* 19/5, *S. marcescens* 19/10, *P. mirabilis* 19/11, *M. morganii* 19/12 and *A. baumannii* 21/7; three strains in which the colistin MIC (4 mg/L) was just above the breakpoint defined by EUCAST (2 mg/L), *E. coli* 21/2, *E. cloacae* 21/4 and *K. pneumoniae* 21/5 [23]; and *P. aeruginosa* ATCC 27853 and *A. baumannii* ATCC 19606.

Time-kill assays were performed following a previously described protocol [16]. Overnight cultures of the eleven strains were inoculated into fresh MHB supplemented with colistin (4 mg/L) alone or in combination with CN at 114 or 57 mg/L and BA at 5.4 × 10^3^ mg/L. The growth of strains in MHB supplemented with CN or BA alone, at the indicated concentrations, and in MHB supplemented with ethanol or DMSO, at the same % (v/v) as CN and BA respectively, was also evaluated. A lower concentration of BA, 2.7 × 10^3^ mg/L, was also assessed with *A. baumannii* 21/7 as this strain was more susceptible to BA. Similarly, MHB without colistin, BA, or CN was inoculated with every strain and used as a control for bacterial growth. All time-kill assays were performed at least three times, and the standard deviations were calculated. The data are presented in graphs as the mean ± standard deviation (SD).

Bactericidal activity for colistin + CN, colistin + BA and CN and BA alone was defined as a decrease of ≥ 3 log_10_ cfu/mL compared to the initial inoculum. Likewise, synergy between colistin + CN and colistin + BA was defined as a decrease of ≥ 2 log_10_ cfu/mL compared to growth in the presence of colistin alone [16].

### Analysis of the mode of action of the CN and BA

To assess whether the ability of CN and BA to reverse resistance to colistin was similar to that described for aspirin (AS), sodium benzoate (SB) and sodium salicylate (SS), the effects of 300 mM sucrose, 1 mM L-arginine and 1 mM L-glutamic acid on the MICs of colistin + CN and colistin + BA were evaluated. Neither sucrose nor amino acids alone inhibited bacterial growth [16].

## Results

### Analysis of colistin resistance

The MICs of colistin ranged from 4 to 4096 mg/L in the fifteen studied colistin-resistant clinical isolates (Tables 1 and 2). No amplification of any of the *mcr* genes was detected by PCR in the studied strains. Similarly, conjugation assays confirmed the lack of transferability of colistin resistance. Taken together, these results indicate that colistin resistance was most likely chromosomally mediated in the studied strains.

**Table 1:** Effects of different concentrations of CN on colistin MICs (mg/L) and influence of L-arginine, L-glutamic acid and sucrose

	CST	CN	CST + CN-29	CST + CN-57	CST + CN-114	CST + CN-57			CST + CN-114		
						+ Arg	+ Glu	+ SC	+ Arg	+ Glu	+ SC
*E. coli* ATCC 25922	0.5	218.5	2	2	0.5	1	0.5	1	1	0.5	0.5
*E. coli* 19/2	8	218.5	4	4	2	1	2	2	1	1	≤ 0.06
*E. coli* 21/1	4	218.5	4	4	1	2	2	2	0.5	1	0.25
*E. coli* 21/2	4	218.5	2	2	0.5	1	1	2	1	1	0.5
*E. cloacae* 19/3	4096	218.5	512	256	0.25	0.5	2	1	0.5	1	4
*E. cloacae* 21/3	4	218.5	4	4	0.5	2	2	2	1	1	1
*E. cloacae* 21/4	4	218.5	2	2	1	2	2	2	2	1	1
*K. pneumoniae* ATCC 700603	1	437	1	1	1	2	1	2	1	1	2
*K. pneumoniae* 19/4	64	218.5	4	4	4	2	4	4	2	2	2
*K. pneumoniae* 19/5	128	437	64	16	2	2	4	2	2	2	1
*K. pneumoniae* 19/6	64	218.5	4	4	2	4	2	2	1	1	1
*K. pneumoniae* 19/7	64	218.5	2	2	1	0.5	1	0.5	2	1	1
*K. pneumoniae* 19/8	64	437	4	4	4	2	2	2	1	1	1
*K. pneumoniae* 21/5	4	218.5	2	2	2	2	2	2	1	2	2
*P. aeruginosa* ATCC 27853	1	874	2	1	1	2	1	2	2	1	2
*A. baumannii* ATCC 15308	2	218.5	2	2	2	1	1	0.5	0.5	0.25	0.5
*A. baumannii* ATCC 19606	2	218.5	2	1	1	1	0.5	1	0.25	≤ 0.06	≤ 0.06
*A. baumannii* 21/6	256	874	256	64	≤ 0.06	16	16	64	≤ 0.06	≤ 0.06	≤ 0.06
*A. baumannii* 21/7	256	218.5	256	64	≤ 0.06	128	128	32	≤ 0.06	≤ 0.06	≤ 0.06
*S. marcescens* 19/10	4096	218.5	128	4	0.25	1	1	1	0.5	0.5	0.5
*P. mirabilis* 19/11	4096	218.5	4096	4	0.5	1	1	1	1	0.5	0.5
*M. morganii* 19/12	4096	218.5	4096	128	0.25	512	4096	4096	64	2	≤ 0.06

*CST *colistin, *CN *cinnamaldehyde, CN-29, 28.5 mg/L CN; CN-57, 57 mg/L CN; CN-114, 114 mg/L CN; Arg, 1 mM L-arginine; Glu, 1 mM L-glutamic acid; SC, 300 mM sucrose

**Table 2:** Effects of different concentrations of BA on colistin MICs (mg/L) and influence of L-arginine, L-glutamic acid and sucrose

	CST	BA	CST + BA-1	CST + BA-3	CST + BA-5	CST + BA-3			CST + BA-5		
						+ Arg	+ Glu	+ SC	+ Arg	+ Glu	+ SC
*E. coli* ATCC 25922	0.5	10,680	2	2	2	0.5	1	0.5	0.5	0.25	≤ 0.06
*E. coli* 19/2	8	10,680	4	4	1	1	1	0.25	1	0.25	≤ 0.06
*E. coli* 21/1	4	10,680	4	4	0.5	1	1	1	1	0.5	0.25
*E. coli* 21/2	4	10,680	4	4	0.5	1	1	0.5	0.5	0.25	≤ 0.06
*E. cloacae* 19/3	4096	10,680	4096	4096	128	64	128	4096	0.25	0.12	512
*E. cloacae* 21/3	4	10,680	8	8	1	1	1	0.5	0.5	0.25	≤ 0.06
*E. cloacae* 21/4	4	10,680	8	4	0.25	1	1	1	0.5	0.25	≤ 0.06
*K. pneumoniae* ATCC 700603	1	10,680	8	4	4	8	8	8	32	64	2
*K. pneumoniae* 19/4	64	10,680	16	16	8	8	32	8	8	16	8
*K. pneumoniae* 19/5	128	10,680	128	128	2	256	256	256	2	4	8
*K. pneumoniae* 19/6	64	10,680	8	4	1	1	1	0.25	2	0.5	≤ 0.06
*K. pneumoniae* 19/7	64	10,680	2	2	≤ 0.06	2	2	2	2	2	≤ 0.06
*K. pneumoniae* 19/8	64	10,680	8	4	2	1	1	0.25	2	1	≤ 0.06
*K. pneumoniae* 21/5	4	10,680	4	4	0.25	8	32	16	4	2	1
*P. aeruginosa* ATCC 27853	1	10,680	4	4	0.25	1	2	2	≤ 0.06	0.25	≤ 0.06
*A. baumannii* ATCC 15308	2	10,680	4	2	1	2	2	4	1	8	≤ 0.06
*A. baumannii* ATCC 19606	2	10,680	4	2	≤ 0.06	2	2	2	≤ 0.06	1	≤ 0.06
*A. baumannii* 21/6	256	10,680	256	128	≤ 0.06	256	256	0.25	≤ 0.06	≤ 0.06	≤ 0.06
*A. baumannii* 21/7	256	5340	256	128	≤ 0.06	256	256	0.06	≤ 0.06	≤ 0.06	≤ 0.06
*S. marcescens* 19/10	4096	10,680	2	0.5	≤ 0.06	2	4	4	≤ 0.06	0.5	0.25
*P. mirabilis* 19/11	4096	10,680	2	2	0.5	1	1	0.25	0.5	0.25	≤ 0.06
*M. morganii* 19/12	4096	10,680	4096	4096	128	> 2048	> 2048	> 4096	≤ 0.06	≤ 0.06	≤ 0.06

*CST *colistin, *BA *baicalin, BA-1, 1.3 × 10^3^ mg/L BA; BA-3, 2.7 × 10^3^ mg/L BA; BA-5, 5.4 × 10^3^ mg/L BA; Arg, 1 mM L-arginine; Glu, 1 mM L-glutamic acid; SC, 300 mM sucrose

### Assessment of the antibacterial activity of potential adjuvants with colistin

The highest concentrations of CN and BA were the most effective at decreasing the MICs of colistin, up to > 68,266-fold decreases (Tables 1 and 2). Furthermore, the two lowest concentrations of CN and BA decreased the MICs of colistin by 2- to 8192-fold, although the lowest CN concentrations were somewhat more effective at decreasing the MICs of colistin in *E. coli* 21/2, *E. cloacae, K. pneumoniae, M. morganii* and *A. baumannii* strains, whereas those of BA were more effective in *S. marcescens* and *P. mirabilis* (Tables 1 and 2).

Similarly, the decreases in the MICs of colistin in the wild-type strains were mainly induced by the highest concentration of BA (Table 2).

FICI analysis revealed synergy between the highest concentrations of CN and BA with colistin in all resistant strains except for colistin + CN in *K. pneumoniae* 21/5. Colistin + CN-114 had no-interaction in the susceptible wild-type strains. The colistin + BA-5 combination was more synergistic than colistin + CN in two *E. coli* and *K. pneumoniae* strains, *S. marcescens*, *P. mirabilis, P. aeruginosa* ATCC 27853 and the *A. baumannii* wild-type strains (Table 3).

**Table 3:** FIC indices and drug interaction of the combinations of colistin with CN and BA at different concentrations

	CST + CN-29		CST + CN-57		CST + CN-114		CST + BA-1		CST + BA-3		CST + BA-5	
	FICI	DI^a^	FICI	DI^a^	FICI	DI^a^	FICI	DI^a^	FICI	DI^a^	FICI	DI^a^
*E. coli* ATCC 25922	4.009	A	4.009	A	1.002	NI	4.000	A	4.000	A	4.000	A
*E. coli* 19/2	0.518	NI	0.518	NI	0.259	S	0.500	S	0.500	S	0.125	S
*E. coli* 21/1	1.018	NI	1.018	NI	0.255	S	1.000	NI	1.000	NI	0.125	S
*E. coli* 21/2	0.505	NI	0.505	NI	0.126	S	1.000	NI	1.000	NI	0.125	S
*E. cloacae* 19/3	2.468	NI	1.234	NI	0.001	S	1.384	NI	1.384	NI	0.043	S
*E. cloacae* 21/3	1.018	NI	1.018	NI	0.127	S	2.001	NI	2.001	NI	0.250	S
*E. cloacae* 21/4	0.509	NI	0.509	NI	0.255	S	2.001	NI	1.000	NI	0.063	S
*K. pneumoniae* ATCC 700603	1.002	NI	1.002	NI	1.002	NI	8.001	A	4.000	A	4.000	A
*K. pneumoniae* 19/4	0.081	S	0.081	S	0.081	S	0.251	S	0.251	S	0.126	S
*K. pneumoniae* 19/5	0.646	NI	0.162	NI	0.020	S	1.012	NI	1.012	NI	0.016	S
*K. pneumoniae* 19/6	0.081	S	0.081	S	0.040	S	0.126	S	0.063	S	0.016	S
*K. pneumoniae* 19/7	0.040	S	0.040	S	0.020	S	0.031	S	0.031	S	0.001	S
*K. pneumoniae* 19/8	0.072	S	0.072	S	0.072	S	0.126	S	0.063	S	0.031	S
*K. pneumoniae* 21/5	0.509	NI	0.509	NI	0.509	NI	1.000	NI	1.000	NI	0.063	S
*P. aeruginosa* ATCC 27853	2.002	NI	1.001	NI	1.001	NI	4.000	A	4.000	A	0.250	S
*A. baumannii* ATCC 15308	1.002	NI	1.002	NI	1.002	NI	2.000	NI	1.000	NI	0.500	S
*A. baumannii* ATCC 19606	1.009	NI	0.505	NI	0.505	NI	2.000	NI	1.000	NI	0.030	S
*A. baumannii* 21/6	3.343	NI	0.836	NI	0.001	S	1.024	NI	1.024	NI	0.000	S
*A. baumannii* 21/7	2.172	NI	0.25	S	0.001	S	1.024	NI	1.024	NI	0.000	S
*S. marcescens* 19/10	0.617	NI	0.019	S	0.001	S	0.001	S	0.000	S	0.000	S
*P. mirabilis* 19/11	19.746	A	0.019	S	0.002	S	0.001	S	0.001	S	0.000	S
*M. morganii* 19/12	19.746	A	0.617	NI	0.001	S	1.384	NI	1.384	NI	0.043	S

*CST *colistin, CN-29, 28.5 mg/L CN; CN-57, 57 mg/L CN; CN-114, 114 mg/L CN; BA-1, 1.3 × 10^3^ mg/L BA; BA-3, 2.7 × 10^3^ mg/L BA; BA-5, 5.4 × 10^3^ mg/L BA; FICI, FIC index; *DI *drug interaction

^a^Drug interaction: S, synergy; NI, no interaction; A, antagonism

### Time-kill assays

**Fig. 1:**
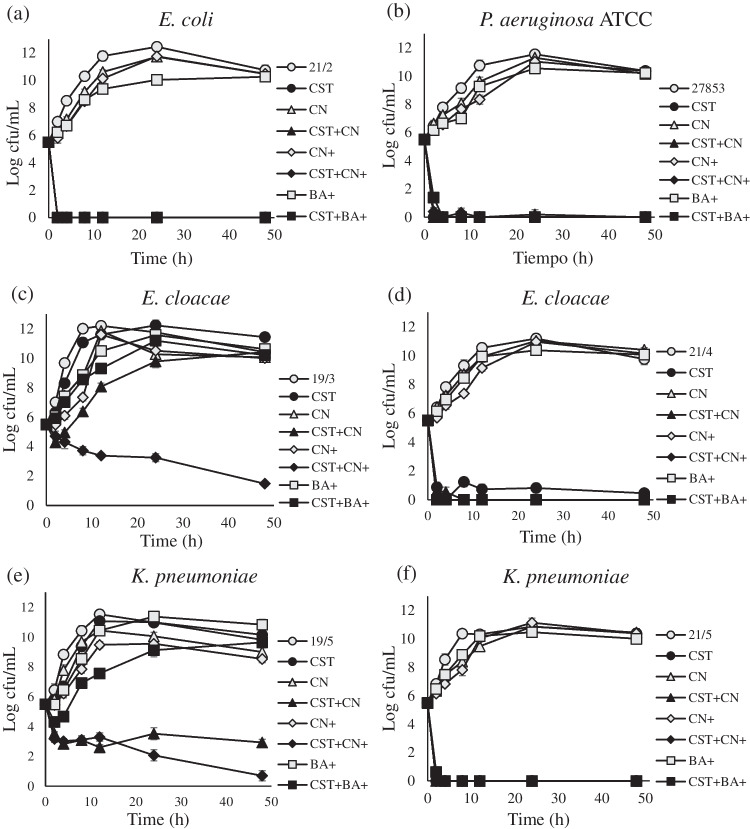

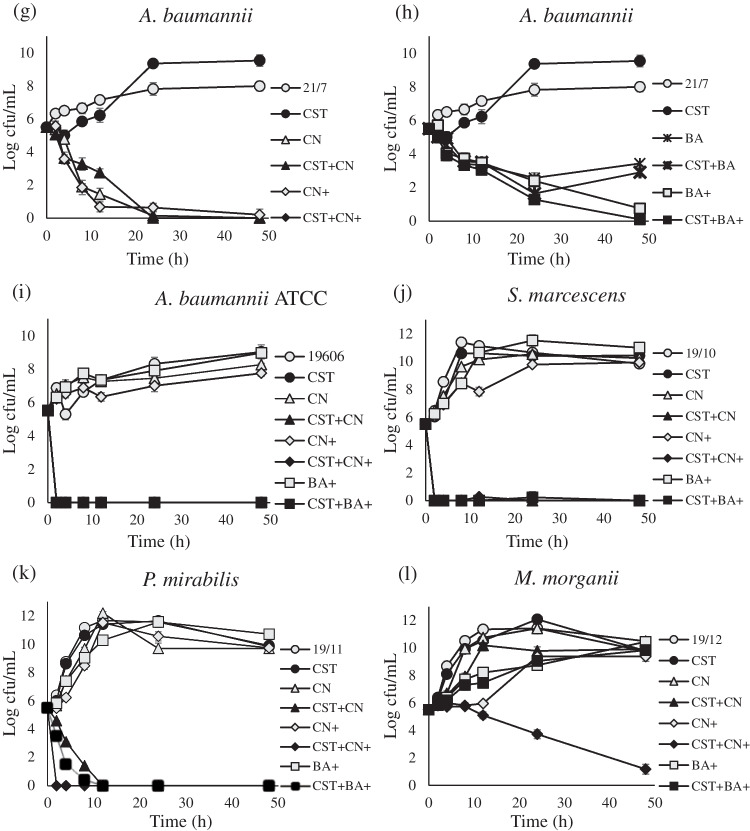
Time kill assays with colistin. Growth (cfu) of strains in Mueller-Hinton broth without colistin nor any chemical (open circle). Growth (cfu) of strains with 4 mg/L CST alone: CST (filled circle). Growth (cfu) of strains with the following combinations of colistin 4 mg/L and chemicals, Cinnamaldehyde-57 mg/L: CN (open triangle), CST + Cinnamaldehyde-57 mg/L: CST + CN (filled triangle), Cinnamaldehyde-114 mg/L: CN+ (open diamond), CST + Cinnamaldehyde-114 mg/L: CST + CN+ (filled diamond), Baicalin-2.7 × 10^3^ mg/L: BA (cross), CST + Baicalin-2.7 × 10^3^ mg/L: CST + BA (asterisk), BA-5.4 × 10^3^ mg/L: BA+ (open square), CST + BA-5.4 × 10^3^ mg/L: CST + BA+ (filled square), The experiments were performed in triplicate, and the error bars represent the standard deviation. The standard deviation values ranged from 0- to 0.47 Log cfu/mL

The presence of 4 mg/L colistin did not generally decrease the growth of strains in which colistin MICs ≥ 128 mg/L by more than 1 log_10_ CFU/mL (Fig. 1). CN or BA had bactericidal effects against only *A. baumannii* 21/7 (Fig. 1g and h). DMSO alone decreased the growth of *A. baumannii* 21/7 by 1-1.6 units but not that of the remaining strains.

The colistin + CN/BA combinations were not more bactericidal than colistin alone in strains in which the colistin MICs were 4 mg/L (Fig. 1a and f), except for *E. cloacae* 21/4 (Fig. 1d). In contrast, the colistin + CN/BA combinations, but not colistin alone, had bactericidal effects against *E. cloacae* 19/3, *K. pneumoniae* 19/5, *A. baumannii* 21/7, *S. marcescens*, *P. mirabilis* and *M. morganii* (Fig. 1c, e, g, h, j, k, l), although colistin + CN had greater bactericidal and synergistic activity than colistin + BA (Fig. 1c, e, g, h and l).

The synergy detected by time-kill assays was consistent with the FICI results for most colistin-resistant *Enterobacterales* (MIC ≥ 128 mg/L), except colistin + CN-57 in *E. cloacae* 19/3 and *K. pneumoniae* 19/5 (Fig. 1c and e) (Table 3). Synergy, in both the time-kill assays and FICI analysis, was concentration-dependent; as the concentrations of CN and BA increased, the activity in combination with colistin became more synergistic.

### Effects of L-arginine, L-glutamic acid and sucrose on the MICs of colistin + CN/BA

L-arginine, L-glutamic acid and sucrose generally decreased the MICs of colistin (data not shown in tables) and colistin + CN/BA by 2- to 4-fold, with some exceptions (Tables 1 and 2). Sucrose decreased the MICs of colistin + BA 8-to 2048-fold in the *E. coli*, *E. cloacae* 21/3, *K. pneumoniae* 19/6 and 19/8, *A. baumannii* and *P. mirabilis* strains (Table 2) and decreased the MICs of colistin + CN 8- to 32-fold in the 19/2, 19/5 and *A. baumannii* ATCC 19606 strains (Table 1). In contrast, L-arginine, L-glutamic acid and sucrose alone did not change the MICs of colistin in *A. baumannii* strains (data not shown in tables). Likewise, the two amino acids and sucrose induced 2- to 4-fold increases in the MICs of colistin in the *E. coli* and *K. pneumoniae* ATCC strains (data not shown in tables) and 2-fold increases in the MICs of colistin + CN/BA in a few strains (Tables 1 and 2). However, they increased the MIC of colistin + CN by 4- to 256-fold in *M. morganii* (Table 1). Similarly, L-arginine, L-glutamic acid and sucrose increased the MICs of colistin + BA 4- to 32-fold in the 19/10, 19/5, 19/7, 21/5, ATCC 700603, ATCC 15308 and ATCC 19606 strains (Table 2).

## Discussion

Compared to the *de novo*- synthesis of new antibiotics, natural substances with proven use in humans that restore colistin susceptibility in MDR bacteria represent an advantageous alternative. In a previous study, we demonstrated that AS, SB and SS reverse colistin-resistance [16]. Herein, we focused on CN and BA, which have antimicrobial, antioxidant, antipyretic and anti-inflammatory properties [11, 26, 27]. Our findings indicated that at 1/2, 1/4 and 1/8 of the MICs of CN and BA, colistin resistance was partially or fully reversed in a concentration-dependent manner in *A. baumannii* and *Enterobacterales*, including the *Proteus* and *Morganella* strains. This effect was similar to that previously described for carbonyl cyanide m-chlorophenylhydrazone (CCCP) [16, 28]. CN and BA might also effectively decrease *mcr*-mediated colistin resistance, as described in a study using CCCP [28]. In this regard, *mcr1*-mediated colistin resistance does not differ from that found in intrinsically resistant gram-negative bacteria [5, 29].

CN was more effective than BA in decreasing the MICs in strains with the highest level of colistin resistance, except for *S. marcescens* and *P. mirabilis*. This finding might be associated with the mildly acidic pH induced by BA, given that an acidic environment has been reported to contribute to polymyxin resistance [5].

CN and BA were less effective at decreasing colistin-MICs in wild-type susceptible strains, possibly because of the induction of efflux pump expression by these flavonoids, as previously described [5, 21, 30]. In contrast, the marked decrease in colistin MICs elicited by CN and BA in resistant strains might have been linked to cell wall damage associated with chromosomally mediated colistin resistance, which would increase the susceptibility of colistin-resistant strains to CN and BA [5, 31]. In fact, LPS loss and decreased outer membrane integrity were observed by Moffatt JH et al. in colistin-resistant mutants derived from *A. baumannii* ATCC 19606 [13]. In support of this hypothesis, the test concentrations of CN and BA were found to be bactericidal in the in vitro selected mutant *A. baumannii* 21/7 but not in its parental wild-type strain, ATCC 19606.

The synergy detected in FIC analyses, confirmed the efficacy of CN and BA as colistin adjuvants. However, colistin + CN was more bactericidal than colistin + BA against the colistin-resistant strains 19/12, 19/3 and 19/5, but lower than that described for AS and SS against 19/3 and 19/5 [16]. The synergy between cinnamaldehyde and polymyxin B against *Enterobacterales* has been associated with protein leakage from bacterial cells [32]. In addition, the rapid bactericidal action of colistin + CN/BA against *S. marcescens*, *P. mirabilis* and *A. baumannii* 21/7 suggests that cell damage might have occurred, as previously reported for combinations of CN with other antimicrobials against *Enterobacterales* [32–34].

The time-kill assay findings also support the efficacy of CN and BA in decreasing the colistin MIC against the most resistant strains, as in the presence of either of the two chemicals, 4 mg/L colistin was enough to inhibit the growth of bacteria in which colistin MICs were ≥ 128 mg/L. Therefore, CN and BA would enable the administration of lower colistin doses and reduce consequent toxicity. Furthermore, the antioxidant and renoprotective effects of CN and BA might prevent or mitigate colistin nephrotoxicity [35–37].

Interestingly, the most effective concentration of CN (114 mg/L) was 47-fold lower than that of BA (5.4 × 10^3^ mg/L), although both were within the reported nontoxic range (up to 0.4 g/kg CN and 600 mg/kg BA). In addition, CN and BA have shown antimicrobial activity at nontoxic concentrations in vivo [8, 12, 38, 39].

Colistin resistance is associated with a decrease in the net charge of lipid A from − 1.5 to 0 in the bacterial outer membrane [40]. Therefore, we assessed L-arginine, L-glutamic acid and sucrose to examine the mode of action of CN and BA, according to a previously described method [16, 41].

L-arginine, L-glutamic acid and sucrose generally induced decreases in the MICs of colistin + CN/BA. This finding might be explained by further inhibition of bacterial growth by these three osmolytes if the bacterial cell walls were somewhat disrupted by CN and BA. Thus, changes in the transmembrane electrical potential were ruled out as an explanation for the reversal of colistin resistance by CN and BA, given that the protonophores sucrose and L-arginine did not restore colistin resistance in the presence of CN and BA, as was also described for SS, AS and SB [16]. Unlike BA, CN does not possess any hydroxyl groups in its structure that would allow for depolarization of the cell wall [42, 43]. Indeed, the largest decreases in colistin + CN/BA MICs were induced by 300 mM sucrose. The increase in surface pressure through the binding of these flavonoids to the external monolayer of lipid membranes might explain why the osmolytes sucrose and L-arginine reduced the MICs of colistin + CN and colistin + BA. In fact, CN and BA have been demonstrated to decrease tolerance to high osmolarity and induce disruption/impairment of the membrane/cell wall and increased permeability, which would allow colistin access to its targets [11, 32, 34, 44–48].

Likewise, the increases in the MICs of colistin + CN/BA caused by amino acids and sucrose in some strains might be associated with heteroresistance to colistin and/or the overexpression of efflux pumps [5, 49–51].

The effectiveness of CN and BA in restoring the sensitivity of gram-negative bacteria to colistin may aid in addressing infections caused by *Enterobacterales* strains that are resistant to colistin and carbapenems.

## Conclusion

CN and BA reversed colistin resistance in a concentration-dependent manner in strains of six different species of *Enterobacterales* and *A. baumannii*. Both of these agents also exhibited good synergy with colistin. Further studies are required to determine the therapeutic safety and efficacy of colistin + CN/BA combinations.

## Data Availability

Not applicable.
